# Improving surveillance of emergency department activity through natural language processing

**DOI:** 10.23889/ijpds.v9i5.1667

**Published:** 2024-09-18

**Authors:** Huw Strafford, Beata Fonferko-Shadrach, W. Owen Pickrell, Jane Lyons, Arron Lacey, Jordan Evans, Gareth John, Jennifer Selby, Ronan Lyons

**Affiliations:** 1 Swansea University; 2 Swansea Bay University Health Board; 3 Cardiff and Vale University Health Board; 4 Digital Health and Care Wales

## Objective

Managers, clinicians and policymakers have limited information on near-real time patterns of attendance at emergency departments (ED). Our aim was to develop a surveillance system through extracting information from the presenting complaint to develop and feed activity dashboards, improve injury surveillance and support research.

We developed a Natural Language Processing (NLP) pipeline using the General Architecture for Text Engineering (GATE) system. The pipeline produced Unified Medical Language System (UMLS) codes from ED presenting complaint records and also implemented the Joint Action on Monitoring Injuries in Europe (JAMIE) Minimum Dataset. We classified complaints into 31 common problem presentations (Coughs, Bleeds, Mental Health, Dizziness...), along with categories from JAMIE (Mechanism of Injury, Location, Activity, Intent…).

## Results

Preliminary results are from a pilot study of 86,416 presenting complaints from Morriston Hospital in 2014.

The most common terms used for grouped symptoms/categories were “Injury” (38.6%, n=28691), “Pain” (22.8%, n=19420), “Unwell” (7.2%, n=6117), “Bleeding” (6.7%, n=5760), and “Shortness of Breath” (3.8%, n=3255).

32.3% of records contained Mechanism of Injury codes, including, falls (15.2%, n=13174), struck by/against (6.9%, n=5960), cuts (5.6%, n=4833), road traffic collisions (2.5%, n=2124) and poisoning (1%, n=900). We are continuing to refine and validate the categories using precision and recall and with comparison with coded diagnoses.

## Conclusions and Implications

NLP can be used to extract valuable additional detailed information from ED data. We will further develop the algorithms for all hospitals in Wales for data from 2023 and design dashboards to produce a near-real time surveillance system.

